# Real-Time Detection and Counting Method for Distant-Water Tuna Based on Improved YOLOv10n-EMCNet

**DOI:** 10.3390/s26072240

**Published:** 2026-04-04

**Authors:** Yuqing Liu, Zichen Zhang, Yuanchen Cheng, Hejun Liang, Jiacheng Wan, Chenye Wang

**Affiliations:** College of Engineering Science and Technology, Shanghai Ocean University, Shanghai 201306, China; yqliu@shou.edu.cn (Y.L.);

**Keywords:** distant-water fisheries, tuna detection, real-time monitoring, object detection, multi-object tracking, automated counting

## Abstract

Reliable real-time detection and counting of tuna during distant-water deck operations is critical for automated catch monitoring but remains challenging due to strong illumination variation, background clutter, and frequent occlusion. This study proposes YOLOv10n-EMCNet, an improved lightweight detector based on YOLOv10n, integrating an ESC-based C2f enhancement in the backbone, a Multi-Branch and Scale Modulation-Fusion Feature Pyramid Network (SMFPN) in the neck, and a Convolutional Attention Fusion Module (CAFM) in the head for fine-grained representation and multi-scale feature fusion. An end-to-end detection–tracking–counting pipeline is further constructed by combining the detector with DeepSORT and an ROI-based de-duplication strategy. On the tuna dataset, YOLOv10n-EMCNet achieved 94.84% mAP@0.5, 65.29% mAP@0.5:0.95, and 91.77% recall with 6.5 GFLOPs. In addition, a controlled comparison among DeepSORT, ByteTrack, and OC-SORT on challenging videos showed that DeepSORT provided the best overall balance between counting accuracy, identity stability, and runtime efficiency. In shipboard video validation on four representative videos covering daytime high glare, nighttime low light, dense occlusion, and dense multi-target, the proposed pipeline achieved an average counting accuracy of 91.4%, with an average relative error of 8.62% and an average absolute error of 1.25 fish per video, while operating at approximately 30 FPS on an RTX 4090D platform. These results provide encouraging preliminary evidence that the proposed method can support automated tuna monitoring under representative shipboard conditions.

## 1. Introduction

Distant-water tuna fisheries play an important role in global seafood supply and national fishery economies. For effective stock assessment and quota enforcement, reliable catch statistics are essential, particularly for high-value species such as albacore and yellowfin tuna [1,2]. In practice, however, catch monitoring on distant-water vessels still relies heavily on human observers and manual recording, which is labor-intensive, costly, and prone to delayed reporting and occasional omissions under long working hours and harsh operating conditions.

Electronic monitoring (EM) systems have therefore been increasingly deployed on fishing vessels to provide continuous video records of deck operations [3,4]. This opens a pathway toward automated, data-driven supervision using computer vision, enabling real-time detection and counting of catches from video streams [5]. Nevertheless, distant-water deck footage poses distinct challenges compared with typical benchmark scenarios: illumination varies drastically (e.g., specular reflections and low-light conditions), motion blur is common due to vessel dynamics, and targets frequently overlap or become partially visible during hauling and conveyance. These factors jointly reduce detection recall and disrupt temporal association, which can ultimately undermine counting reliability.

With the rapid progress of deep learning, CNN-based object detectors have become the dominant approach for fish-related visual tasks. Several studies have improved YOLO-series detectors by integrating attention mechanisms or enhancing feature fusion, reporting gains in complex aquatic environments [6,7]. Other efforts have explored fish species recognition and operational event detection using EM data on fishing vessels [8,9]. For counting applications, combining detection with multi-object tracking (MOT) is a common practice; DeepSORT [10] and ByteTrack [11] have been adopted in fish tracking and counting pipelines [12,13]. Despite these advances, two limitations remain for distant-water deck monitoring. First, many existing models are developed and validated primarily in underwater or relatively controlled conditions [6,7], and their robustness can degrade in deck scenes dominated by clutter, reflections, and dense occlusion. Second, for practical shipboard deployment, models must achieve a strong accuracy–efficiency balance on resource-limited hardware platforms, where overly compressed feature representations may lead to missed detections in challenging interference conditions [14].

To address these issues, we propose a real-time detection and counting method for distant-water tuna based on an improved YOLOv10n detector. Taking YOLOv10n [15] as the baseline, we develop an enhanced detector, termed YOLOv10n-EMCNet, tailored for deck scenarios with dense occlusion and strong environmental disturbances. The proposed design improves fine-grained representation and multi-scale interaction by integrating (i) an ESC-based C2f module in the backbone [16], (ii) a Multi-Branch and Scale Modulation-Fusion Feature Pyramid Network (SMFPN) in the neck inspired by FPN [17], and (iii) a Convolutional Attention Fusion Module in the head [18]. Based on the improved detector, we further construct an end-to-end detection–tracking–counting pipeline by coupling DeepSORT [10] with an ROI-based de-duplication strategy to reduce repeated counting caused by target re-entry and boundary truncation.

The main contributions of this work are summarized as follows:

- We propose YOLOv10n-EMCNet, a lightweight detector tailored for distant-water tuna deck monitoring, improving robustness to background clutter, illumination variation, and dense occlusion.
- We design and integrate a scale-modulated multi-branch feature pyramid and a local–global attention fusion head to strengthen multi-scale representation and anti-interference feature fusion in challenging deck scenes.
- Building upon the proposed YOLOv10n-EMCNet detector, we further construct an end-to-end detection–tracking–counting pipeline with multi-object tracking and ROI-based de-duplication counting, enabling category-wise catch statistics from continuous EM video streams.

## 2. Materials and Methods

### 2.1. Data Source and Preprocessing

**Figure 1:**
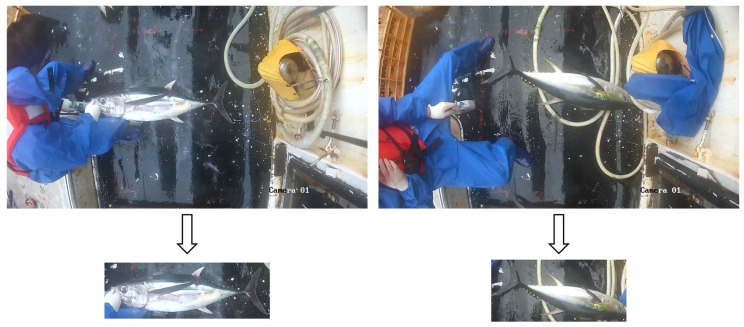
Typical sample images of the studied tuna species.

The experimental data were collected during a complete production voyage of the distant-water tuna longline vessel *Zhongshui 747* operated by China National Fisheries Corporation (CNFC) in the high seas of the South Pacific Ocean. A Hikvision DS-2SC3Q120MY-TE industrial camera (Hikvision, Hangzhou, China) was installed in the bow working area to continuously record catch retrieval, conveyance, and handling. The monitoring targets were albacore tuna and yellowfin tuna, as shown in Figure 1.

After annotation and screening, 2350 images were retained, including 1794 albacore and 556 yellowfin samples. The original dataset was first divided into training and validation subsets at an 8:2 ratio using stratified sampling. Data augmentation was then applied only to the training subset, whereas the validation subset remained unchanged and contained only original images. Therefore, no augmented samples derived from the same original image appeared in both subsets. The augmentation strategy was designed to alleviate the class imbalance in the original dataset [19], particularly the underrepresentation of yellowfin tuna, and to improve model robustness under challenging deck conditions such as illumination variation, rain/fog interference, and image noise. The applied augmentation operations included random rotation, flipping, scaling, Gaussian noise, salt-and-pepper noise, and brightness/contrast adjustment. After augmentation, the combined dataset used in the experiments consisted of 3462 images in total, including the augmented training subset and the original, non-augmented validation subset; across these two subsets, 1794 images were albacore and 1668 were yellowfin. It should also be noted that the image dataset was not constructed by densely sampling consecutive frames from continuous videos. Instead, representative tuna images were first captured from the shipboard videos and then manually screened to remove highly similar samples, thereby reducing temporal redundancy and increasing sample diversity in terms of position, pose, and visible state.

### 2.2. Improved YOLOv10n-EMCNet Detector

**Figure 2:**
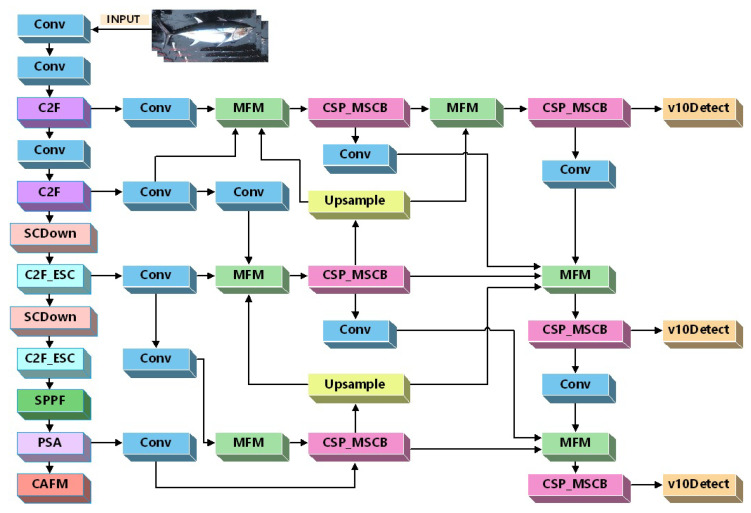
Overall network architecture of the improved YOLOv10n-EMCNet.

To address background clutter, severe occlusion, and multi-scale variation in distant-water deck scenarios, we propose YOLOv10n-EMCNet based on YOLOv10n [15]. “EMC” denotes three introduced components: (i) ESC (Emulating Self-attention with Convolution) embedded in the backbone to enhance fine-grained representation [16]; (ii) a Multi-Branch and Scale Modulation-Fusion Feature Pyramid Network (SMFPN) in the neck to strengthen multi-scale interaction [17]; and (iii) a Convolutional Attention Fusion Module (CAFM) in the head to improve robustness against environmental disturbances [18]. As illustrated in Figure 2, the proposed model preserves the baseline “backbone–neck–head” topology while improving adaptability to complex deck conditions.

#### 2.2.1. ESC-Based C2f Module

**Figure 3:**
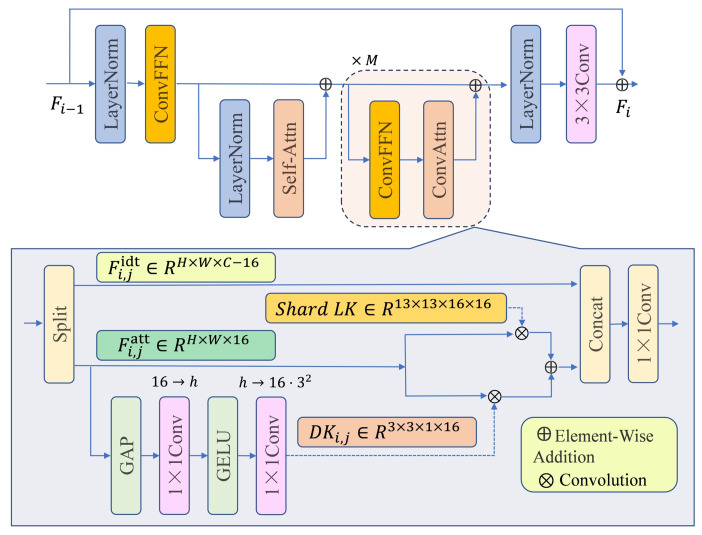
Structure of the C2f module based on emulating self-attention with convolution (ESC).

In stacked and heavily occluded scenes, the original C2f module in YOLOv10n may be limited by locality. We therefore reconstruct C2f by incorporating ESC (Figure 3). ESC combines large-kernel convolution with dynamic convolution to approximate long-range dependency modeling at low cost [16]. Concretely, features are processed by a convolutional feed-forward network and split into an attention branch and an identity branch; the attention branch uses global average pooling and an MLP to generate dynamic kernels, which operate together with a shared large kernel to enable adaptive weighting across space and channels [20]. This design strengthens sensitivity to subtle cues (e.g., boundaries and fin structures), improving discrimination under partial visibility.

#### 2.2.2. Multi-Branch and Scale Modulation-Fusion Feature Pyramid Network

**Figure 4:**
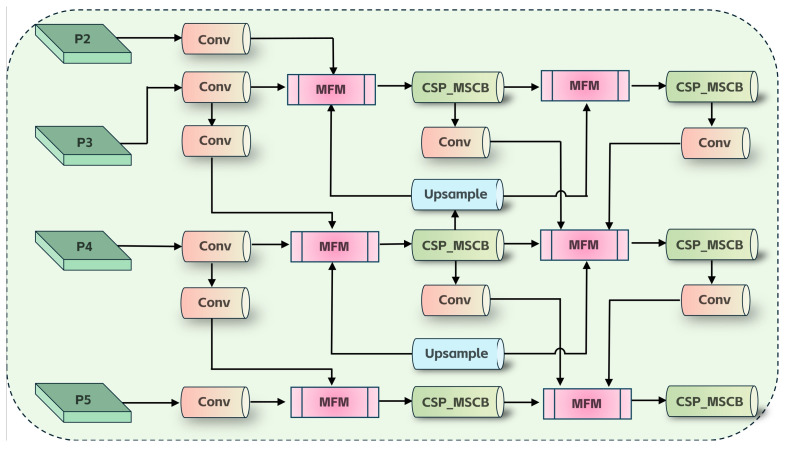
Structure of SMFPN.

Standard FPN-style designs [17] may be insufficient to prioritize informative cues and exploit complementary multi-scale information in cluttered deck environments. We therefore introduce SMFPN into the neck of YOLOv10n (Figure 4), integrating lightweight fusion, scale-aware modulation, efficient convolution, and global heterogeneous kernel selection to enhance interaction between shallow detailed features and deep semantic features.

**Figure 5:**
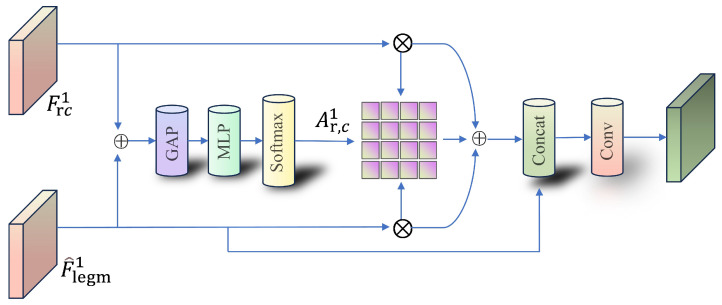
Structure of the Modulation Fusion Module (MFM). ⊕ and ⊗ denote element-wise addition and element-wise multiplication, respectively.

Modulation Fusion Module. To improve cross-scale fusion, a Modulation Fusion Module (MFM) replaces conventional concatenation (Figure 5). MFM applies channel attention [21]: features from two branches are refined and concatenated, then reweighted by attention derived from global pooling and an MLP, and finally integrated by convolution. This modulation better preserves target-relevant channels while suppressing background-induced activations.

**Figure 6:**
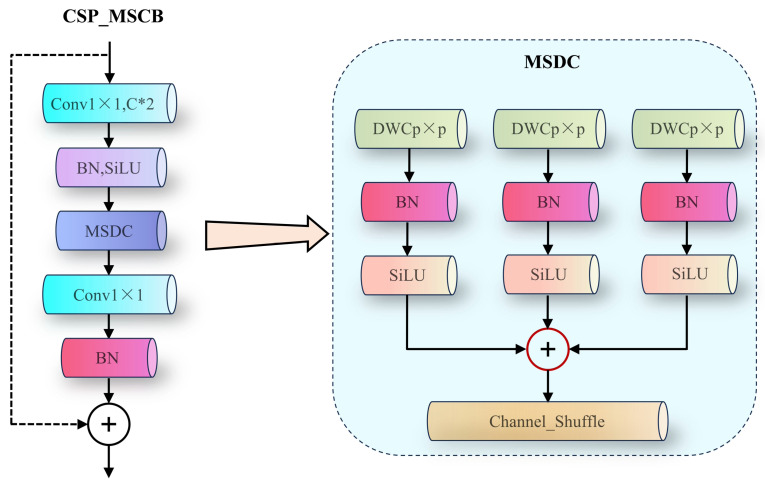
Structure of the efficient multi-scale convolution block and depthwise convolution.

Efficient Multi-Scale Convolution Block. To balance capacity and efficiency, SMFPN incorporates an efficient multi-scale convolution block (CSP_MSCB) (Figure 6). Its core operator, multi-scale depthwise convolution (MSDC), uses three parallel depthwise separable kernels at different scales to capture hierarchical receptive fields [22]. Channel shuffle [23] is further adopted to enhance inter-channel mixing, reducing computation while improving feature diversity for complex backgrounds and scale variation.

Global Heterogeneous Kernel Selection. SMFPN further introduces a Global Heterogeneous Kernel Selection (GHSK) mechanism inspired by dynamic attention concepts [24]. Smaller kernels are emphasized for high-resolution shallow features to preserve edges and textures, whereas larger kernels are favored for deeper features to capture broader context, mitigating the limitations of fixed-kernel configurations.

#### 2.2.3. Convolutional Attention Fusion Module

**Figure 7:**
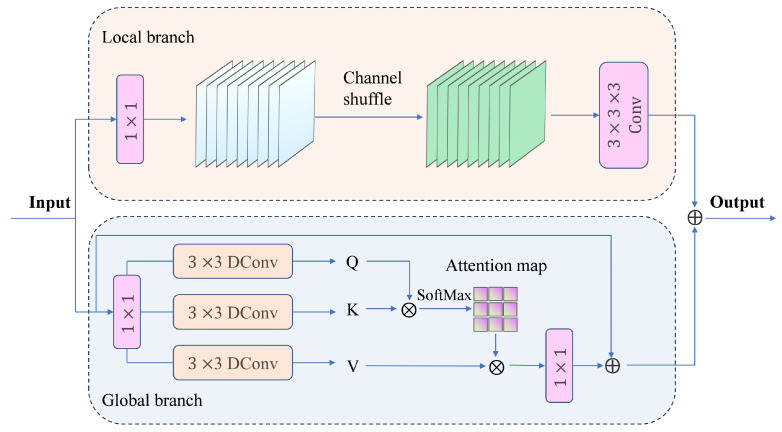
Structure of the Convolutional Attention Fusion Module (CAFM). ⊕ and ⊗ denote element-wise addition and matrix multiplication, respectively.

In deck monitoring, tuna targets are frequently affected by specular reflections, water stains, and dense stacking. We therefore introduce CAFM (Figure 7) [18] to jointly model local details and global dependencies. CAFM uses a dual-branch design: a local branch (channel shuffle and 3×3 convolution) enhances contour/texture details, while a global branch based on self-attention [24] models long-range relations via *Q*, *K*, and *V*. The two branches are fused by element-wise addition, improving detection of overlapping and occluded targets.

### 2.3. Multi-Object Tracking and Counting Based on DeepSORT

To enable continuous monitoring and resolve cross-frame identity ambiguity, we construct a “detection–tracking–counting” pipeline. YOLOv10n-EMCNet provides detections, DeepSORT [10] performs multi-object association, and ROI-based spatiotemporal constraints are applied for reliable counting.

#### 2.3.1. Algorithm Adaptation and Parameter Optimization

DeepSORT combines Kalman-filter motion prediction with cascaded matching using Re-ID embeddings and the Mahalanobis distance [10]. We use the original lightweight CNN in DeepSORT as the appearance feature extractor, rather than reusing detection features from YOLOv10n-EMCNet, to maintain a decoupled embedding space for association. The maximum track retention threshold max_age was set to 30 frames (approximately 1 s) to tolerate short-term occlusion and reduce ID switches, while the track initialization threshold n_init was set to 3 frames to filter transient false detections induced by wave splashes or abrupt illumination changes.

#### 2.3.2. ROI-Based De-Duplication Counting Mechanism

To reduce duplicate counting caused by boundary truncation and targets lingering in the scene, an ROI-based dual verification strategy is adopted. The ROI is defined as 25–75% of the image width and 20–80% of the image height to exclude boundary regions and non-operational areas. A count is triggered only when the geometric center $\left(c_{x},c_{y}\right)$ of a tracked target enters the ROI from outside for the first time and its tracking ID is absent from the counted hash set. This joint constraint on spatial validity and identity uniqueness ensures each tuna is counted at most once within a single operational flow.

### 2.4. Experimental Setup

All experiments were performed on a workstation with Ubuntu 22.04, an Intel^®^ Xeon^®^ Platinum 8481C CPU (16 vCPUs), and an NVIDIA RTX 4090D GPU (24 GB). The software stack included Python 3.9, PyTorch 2.2.2, and CUDA 12.1. Input images were resized to 640×640. The optimizer was SGD with an initial learning rate of 0.01, momentum of 0.937, and weight decay of 0.0005. The batch size was 32 with 8 data-loader workers. All models were trained for 300 epochs under identical settings. Early stopping was enabled when validation performance did not improve for 100 epochs to ensure a fair comparison across methods.

## 3. Results

### 3.1. Evaluation Metrics

To comprehensively evaluate the proposed method for shipboard tuna monitoring, we considered detection, counting, and runtime-related metrics.

For object detection, we report Precision (*P*), Recall (*R*), Average Precision (AP), and mean Average Precision (mAP) [25]. Based on the IoU-based matching criterion, detections are categorized into true positives (TP), false positives (FP), and false negatives (FN). Precision and recall are defined as

(1) $$P=\frac{\mathrm{TP}}{\mathrm{TP}+\mathrm{FP}},\;R=\frac{\mathrm{TP}}{\mathrm{TP}+\mathrm{FN}}.$$

Average Precision (AP) is computed as the area under the precision–recall curve:

(2) $$AP=\int_{0}^{1}P\left(R\right)\;dR,$$

and mean Average Precision (mAP) is defined as

(3) $$mAP=\frac{1}{N}\sum_{i=1}^{N}AP_{i},$$

where *N* denotes the number of categories.

For video-based counting evaluation, we report counting accuracy, relative error, and absolute error. Let $C_{\mathrm{sys}}$ and $C_{\mathrm{gt}}$ denote the system count and manual ground-truth count, respectively. These metrics are defined as

(4) $$\mathrm{Accuracy}=\left(1-\frac{|C_{\mathrm{sys}}-C_{\mathrm{gt}}|}{C_{\mathrm{gt}}}\right)\times 100\%,$$

(5) $$\mathrm{Relative}\;\mathrm{Error}=\frac{|C_{\mathrm{sys}}-C_{\mathrm{gt}}|}{C_{\mathrm{gt}}}\times 100\%,$$

(6) $$\mathrm{Absolute}\;\mathrm{Error}=|C_{\mathrm{sys}}-C_{\mathrm{gt}}|.$$

For tracker comparison, we additionally report end-to-end processing speed in frames per second (FPS) and the number of ID switches, where an ID switch denotes an event in which the same target is assigned different trajectory identities during tracking.

In addition to accuracy metrics, we also report model parameters and GFLOPs to characterize computational complexity, which is important for practical shipboard deployment.

### 3.2. Benchmarking Against Mainstream Lightweight Detectors

**Figure 8:**
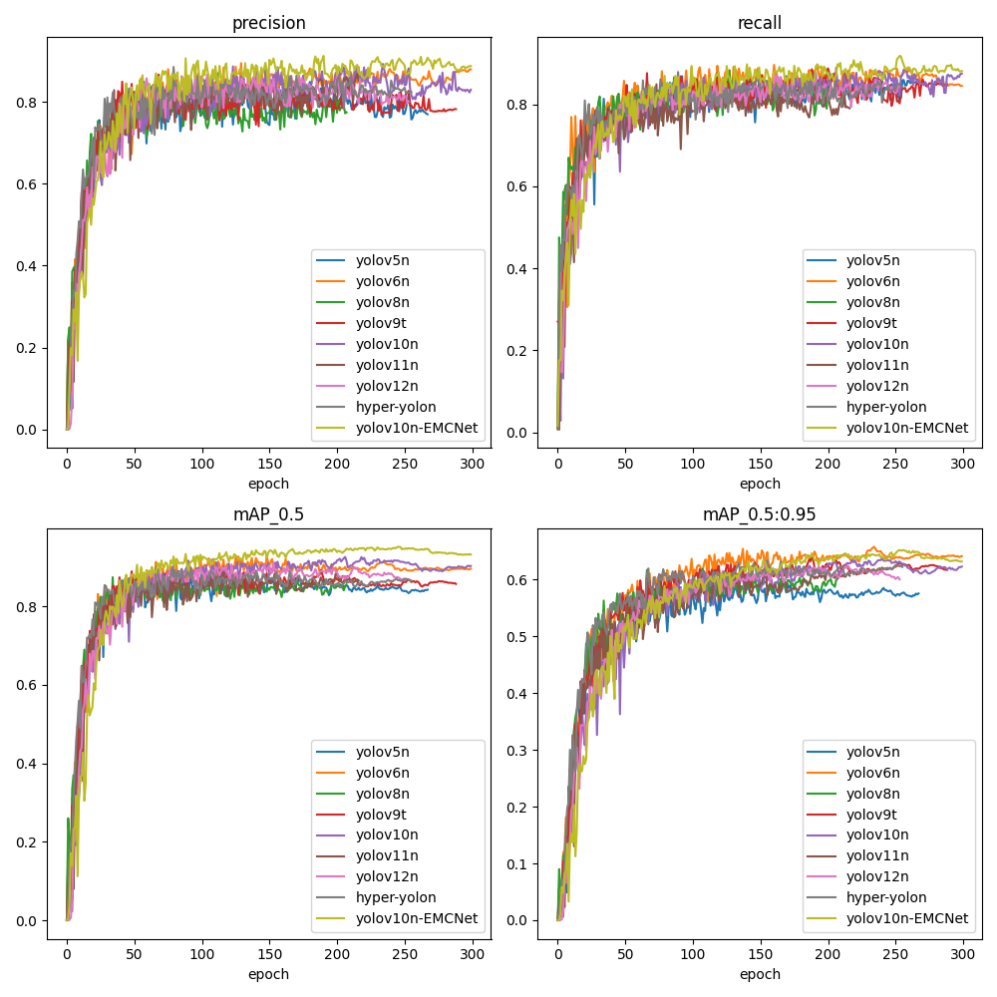
Training metric curves of different models.

To validate the effectiveness of the proposed YOLOv10n-EMCNet, we compared it with representative lightweight detectors, including YOLOv5n [26], YOLOv6n [27], YOLOv8n [28], YOLOv9t [29], YOLOv10n [15], YOLOv11n [30], YOLOv12n [31], and Hyper-YOLOn [32], using the same dataset and training protocol. Unless otherwise specified, the quantitative results reported in Table 1 and Table 2 were evaluated on the original, non-augmented validation subset. Quantitative results are summarized in Table 1, and the training dynamics of the compared models, including the loss and accuracy-related curves, are shown in Figure 8.

Overall, YOLOv10n-EMCNet achieved the strongest accuracy–efficiency trade-off on the distant-water deck dataset. In particular, YOLOv10n-EMCNet reached an mAP@0.5 of 94.84% and a recall of 91.77%. While YOLOv12n reduced computation (5.8 GFLOPs), its performance degraded in the deck environment, with an mAP@0.5 of 91.21% and recall of 82.62%. This gap is operationally meaningful: in automated catch monitoring, missed detections directly translate into under-counting and reduced credibility of statistics, making recall a primary requirement. The results indicate that the proposed design is better aligned with the visual characteristics of deck scenarios (e.g., strong reflections, background clutter, and dense overlap) than general-purpose lightweight detectors primarily optimized for generic benchmarks.

**Figure 9:**
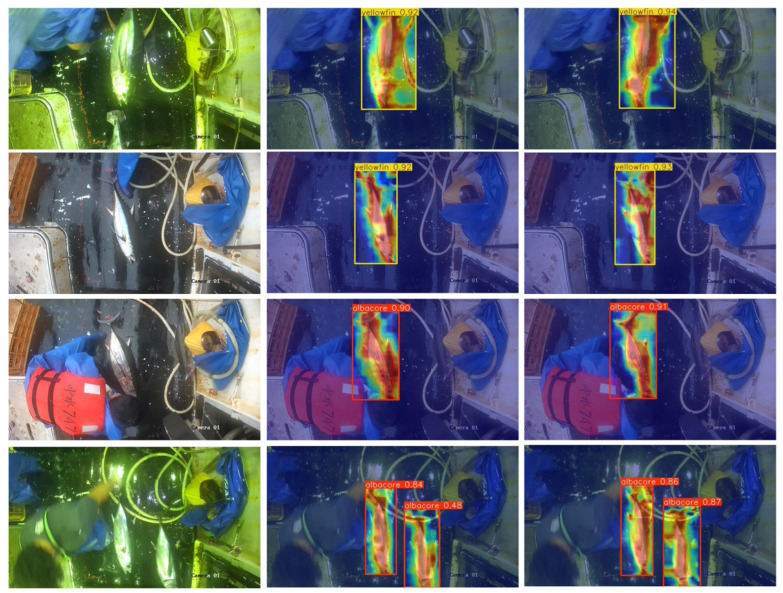
Feature visualization comparison based on Grad-CAM++. Columns from left to right correspond to the original images, YOLOv10n, and YOLOv10n-EMCNet. Warmer colors indicate regions with stronger activation responses, whereas cooler colors indicate weaker responses.

For qualitative comparison, Grad-CAM++ [33] visualizations of YOLOv10n and YOLOv10n-EMCNet are shown in Figure 9. Compared with the baseline YOLOv10n, YOLOv10n-EMCNet exhibits more concentrated and spatially complete responses on tuna targets, while suppressing irrelevant activations in complex deck backgrounds. In challenging cases involving strong reflections, low illumination, partial occlusion, and dense overlap, the baseline model tends to produce more scattered attention, with responses extending to surrounding clutter or failing to fully cover the discriminative target regions. By contrast, the proposed model focuses more consistently on the fish body and boundary-relevant regions, leading to clearer localization of partially visible individuals and more precise separation from nearby interfering structures. This qualitative behavior is consistent with the quantitative improvements reported in Table 1, particularly the gains in mAP@0.5 and recall, and further supports the effectiveness of the proposed detector for complex shipboard tuna monitoring.

### 3.3. Ablation Study

To examine the contribution of each proposed component, we conducted an ablation study by progressively integrating C2f-ESC, SMFPN, and CAFM into the YOLOv10n baseline. The results are reported in Table 2. Unless otherwise specified, the ablation results were also evaluated on the original, non-augmented validation subset.

Adding C2f-ESC improved mAP@0.5:0.95 by 1.51 percentage points with limited computational overhead, suggesting that enhanced fine-grained representation is beneficial for partially occluded tuna instances and boundary localization. SMFPN and CAFM further improved multi-scale interaction and local–global feature fusion, respectively. Notably, when modules were introduced individually, recall showed a slight decrease in some settings, suggesting that isolated module enhancement may shift the precision–recall balance under strong interference. When all three components were integrated, YOLOv10n-EMCNet achieved the best overall performance. Compared with the baseline, mAP@0.5 increased from 91.69% to 94.84%, i.e., by 3.15 percentage points, and recall improved to 91.77%. These results indicate a complementary synergy across the backbone (detail enhancement), neck (scale modulation), and head (robust fusion), which collectively improves reliability in the challenging deck environment.

### 3.4. Comparison of Tracking Methods for Counting Stability

Since counting reliability in the proposed pipeline depends not only on detection performance but also on identity continuity across frames, we further compared three representative tracking methods, namely DeepSORT, ByteTrack, and OC-SORT, under the same detector and counting settings. In this experiment, YOLOv10n-EMCNet was fixed as the detector, and the ROI-based de-duplication counting strategy remained unchanged. Two challenging shipboard videos, corresponding to dense occlusion and dense multi-target, were selected for controlled comparison. The evaluated metrics included counting accuracy, relative error, absolute error, end-to-end FPS, and ID switches, so as to assess the influence of the tracking module on counting stability.

The comparison results are summarized in Table 3. DeepSORT achieved the best overall trade-off among the three tested trackers. Although ByteTrack provided the highest runtime speed, it also exhibited the largest number of ID switches and the largest counting deviations on both challenging videos. OC-SORT showed intermediate performance, but its identity continuity remained less stable than that of DeepSORT. In contrast, DeepSORT achieved the highest average counting accuracy and the lowest ID-switch level while still maintaining real-time processing capability.

It is worth noting that although OC-SORT achieved the same counting accuracy as DeepSORT in the dense multi-target video, it still generated substantially more ID switches, indicating weaker identity continuity under crowded conditions. This suggests that similar final counts do not necessarily imply equally reliable tracking behavior. Overall, the results indicate that reducing ID switches is important for improving ROI-based counting stability in complex shipboard scenarios. Therefore, DeepSORT was selected as the tracking module for the final end-to-end validation of the proposed system.

### 3.5. End-to-End Validation on Shipboard Videos

After selecting DeepSORT as the most suitable tracker through the controlled comparison above, we further evaluated the complete detection–tracking–counting pipeline on four representative shipboard videos covering daytime high glare, nighttime low light, dense occlusion, and dense multi-target. The results are summarized in Table 4.

The proposed system achieved perfect counting consistency with the manual annotation under daytime high-glare conditions. Under more challenging conditions, the counting accuracy was 92.3% in the nighttime low-light scenario, 85.7% in the dense-occlusion scenario, and 87.5% in the dense multi-target scenario. Across the four representative test videos, the total manual count was 54 fish and the total system count was 53, while the system achieved an average counting accuracy of 91.4%, with an average relative error of 8.62% and an average absolute error of 1.25 fish per video. These results provide encouraging preliminary evidence that the proposed pipeline can support automated tuna counting under representative shipboard conditions.

**Figure 10:**
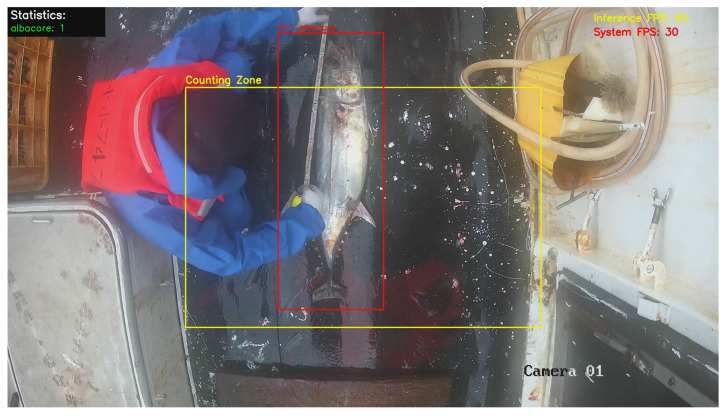
Real-time monitoring results on shipboard operation video.

We further evaluated runtime performance and system-level visualization results (Figure 10). On RTX 4090D, the detector inference speed was approximately 65 FPS (∼15 ms per frame), demonstrating the efficiency of the lightweight architecture. After integrating DeepSORT tracking, ROI-based verification, and video stream visualization/writing, the end-to-end pipeline operated stably at approximately 30 FPS, showing real-time processing capability on a high-performance GPU platform.

## 4. Discussion

### 4.1. Interpretation of Performance Gains in Deck Monitoring

Distant-water deck monitoring differs substantially from common benchmark settings: strong illumination variation (specular reflections and low-light conditions), motion blur due to vessel dynamics, and frequent dense occlusion caused by stacked fish bodies can simultaneously occur. In such scenarios, the dominant failure modes are often boundary ambiguity and partial visibility rather than pure category confusion. The proposed YOLOv10n-EMCNet improves robustness through coordinated enhancements across the detection pipeline. The ESC-based design strengthens fine-grained feature representation under occlusion; SMFPN enhances cross-scale interaction to better preserve partially visible targets; and CAFM integrates local detail sensitivity with global dependency modeling, which is particularly helpful when structured background clutter (e.g., deck, ropes, and water stains) introduces confusing textures. The ablation study suggests that these components are complementary: although individual modules may perturb the precision–recall balance under interference, their joint integration improves recall while maintaining high mAP, which is a critical requirement for reliable catch monitoring.

### 4.2. Detector–Tracker Coupling and Failure Modes Under Dense Occlusion

In an end-to-end “detection–tracking–counting” system, detector recall is tightly coupled with tracking continuity and, consequently, counting reliability. Missed detections fragment trajectories, increase the probability of track termination, and amplify identity re-assignment, which can propagate to duplicate counting when a re-initiated track re-enters the counting ROI. The higher recall achieved by YOLOv10n-EMCNet (91.77%) therefore provides a practical downstream benefit: fewer detection gaps reduce Kalman filter drift and improve the success rate of appearance-based association in DeepSORT, resulting in more stable identities and more reliable counting across typical deck operations.

This interpretation is further supported by the controlled comparison in Section 3.4. Under the same detector and ROI-based counting settings, DeepSORT achieved the best overall balance among the three tested trackers, with an average counting accuracy of 86.6%, an average relative error of 13.40%, an average absolute error of 2.0 fish, an average processing speed of 41.1 FPS, and the lowest average ID-switch level of 2.5. In contrast, ByteTrack provided the highest runtime speed but produced substantially more ID switches and larger counting deviations, while OC-SORT showed intermediate performance. These results suggest that counting stability in challenging deck scenes depends not only on detector quality, but also strongly on the selected tracking strategy.

Nevertheless, dense occlusion and dense multi-target remain the most challenging conditions in the current video-based validation, where the counting accuracy decreases to 85.7% and 87.5%, respectively (Table 4). Two factors are particularly influential. First, when multiple tuna overlap heavily in crowded deck scenes, post-processing (e.g., NMS) may suppress valid boxes for partially visible individuals, producing intermittent detections. Such intermittency can cause DeepSORT to terminate tracks after exceeding the retention threshold (max_age); subsequent re-detection may initiate a new track with a new ID. In edge cases, repeated ROI-entry events associated with re-assigned IDs can still trigger duplicate counting. Second, prolonged occlusion combined with abrupt appearance changes induced by vessel motion and handling operations can degrade the stability of Re-ID embeddings, increasing matching ambiguity and the risk of identity drift. These observations suggest that further improving occlusion robustness—for example, by incorporating occlusion-aware suppression strategies and stronger association mechanisms—is key to enhancing counting reliability under extreme deck conditions.

### 4.3. Limitations and Future Work

Several limitations deserve attention. First, extreme dense occlusion and dense multi-target can still cause intermittent detections and ID re-assignment, ultimately affecting counting stability. Future work could enhance occlusion robustness by adopting occlusion-aware assignment strategies (e.g., soft suppression variants) and/or incorporating temporal consistency constraints. Second, DeepSORT currently uses a lightweight independent CNN for appearance embedding; training a domain-adapted Re-ID network on deck-specific fish instances may improve identity stability in low-light and occlusion scenarios. Third, although the proposed method performs well on the collected voyage data, the current video-based validation remains limited in scale. Broader generalization across vessels, camera viewpoints, sea states, and operational procedures should therefore be validated via multi-voyage and multi-region datasets.

Overall, these results indicate that the proposed YOLOv10n-EMCNet and the associated end-to-end pipeline offer a promising and efficient approach for automated tuna detection and counting under representative shipboard conditions, while also highlighting clear directions for improving robustness under extreme occlusion.

## 5. Conclusions

This study proposed a high-performance lightweight detector, YOLOv10n-EMCNet, for distant-water tuna deck monitoring, and further demonstrated its practical applicability through an integrated detection–tracking–counting pipeline. By reconstructing the backbone with C2f-ESC, optimizing the neck using SMFPN, and enhancing feature fusion with CAFM, the proposed model improved fine-grained representation and multi-scale perception. With only a computational cost of 6.5 GFLOPs, YOLOv10n-EMCNet achieved an mAP@0.5 of 94.84% and demonstrated strong robustness throughout day–night operational cycles, particularly under severe occlusion and pronounced illumination variations. In addition, the end-to-end pipeline based on the improved detector provided encouraging preliminary evidence for automated tuna counting under representative shipboard conditions.

Despite these encouraging results, the current dataset is still limited in terms of acquisition season, fishing area, and video scale, and the stability of the proposed pipeline under extremely adverse weather conditions still requires further validation. Future work will focus on expanding the dataset with samples collected under more diverse seasons, fishing regions, and extreme weather conditions, improving environmental generalization through transfer learning, strengthening counting stability under severe occlusion and dense target overlap, and evaluating inference efficiency on low-power edge computing platforms for future practical deployment.

## Figures and Tables

**Table 1:** Performance comparison of different detection models on the tuna dataset.

Model	mAP@0.5	mAP@0.5:0.95	Precision	Recall	GFLOPs	Parameters
	(%)	(%)	(%)	(%)		
YOLOv5n	87.84	59.96	81.14	84.49	7.10	2,503,334
YOLOv6n	91.30	65.70	87.00	87.27	11.80	4,233,942
YOLOv8n	88.31	61.85	80.51	84.24	8.10	3,006,038
YOLOv9t	89.24	64.34	79.66	88.53	7.60	1,971,174
YOLOv10n	91.69	63.59	82.14	87.10	6.50	2,265,558
YOLOv11n	90.29	61.74	83.01	82.16	6.30	2,582,542
YOLOv12n	91.21	62.50	81.52	82.62	5.80	2,508,734
Hyper-YOLOn	88.79	63.52	81.53	85.47	10.80	3,942,454
YOLOv10n-EMCNet	94.84	65.29	87.48	91.77	6.50	2,661,151

**Table 2:** Results of ablation study on the impact of improved modules.

Test	C2f-ESC	SMFPN	CAFM	mAP@0.5	mAP@0.5:0.95	Precision	Recall
				(%)	(%)	(%)	(%)
1	–	–	–	91.69	63.59	82.14	87.10
2	✓	–	–	91.81	65.10	86.67	88.10
3	–	✓	–	92.95	63.73	87.12	86.60
4	–	–	✓	92.39	63.58	87.50	82.80
5	✓	✓	–	93.92	64.21	90.29	86.71
6	✓	✓	✓	94.84	65.29	87.48	91.77

Note: ✓ indicates that the corresponding module is included, while – indicates that the module is not used.

**Table 3:** Comparison of tracking methods for tuna counting.

Tracker	Avg. Accuracy	Avg. Relative Error	Avg. Absolute Error	Avg. FPS	Avg. ID Switches
	(%)	(%)			
DeepSORT	86.6	13.40	2.0	41.1	2.5
ByteTrack	80.0	20.09	3.0	56.7	8.0
OC-SORT	83.1	16.96	2.5	50.0	6.5

Note: The comparison was conducted on two challenging videos (Video 03: Dense Occlusion; Video 04: Dense Multi-Target). YOLOv10n-EMCNet and the ROI-based counting strategy were fixed for all three trackers.

**Table 4:** End-to-end counting performance of the proposed detection–tracking–counting pipeline on representative shipboard videos.

Video ID	Scenario	Manual Count	System Count	Accuracy	Relative Error	Absolute Error
				(%)	(%)	
01	Daytime (High Glare)	11	11	100.0	0.00	0
02	Nighttime (Low Light)	13	12	92.3	7.69	1
03	Dense Occlusion	14	12	85.7	14.29	2
04	Dense Multi-Target	16	18	87.5	12.50	2
Total	–	54	53	–	–	5
Average	–	13.5	13.25	91.4	8.62	1.25

Note: Manual Count denotes the fish count obtained by frame-by-frame human inspection of each test video.

## Data Availability

The data presented in this study are available upon reasonable request from the corresponding author. The data are not publicly available due to confidentiality and data-use restrictions associated with shipboard operation videos.

## Abbreviations

CAFM	Convolutional Attention Fusion Module
ESC	Emulating Self-attention with Convolution
FPN	Feature Pyramid Network
GFLOPs	Giga Floating-Point Operations
Grad-CAM++	Gradient-weighted Class Activation Mapping++
IoU	Intersection over Union
mAP	mean Average Precision
SMFPN	Multi-Branch and Scale Modulation-Fusion Feature Pyramid Network
MOT	Multi-Object Tracking
Re-ID	Re-Identification
ROI	Region of Interest
